# Analysis of public opinion on employment issues using a combined approach: a case study in China

**DOI:** 10.1038/s41598-024-52158-5

**Published:** 2024-01-23

**Authors:** Chang-Feng Chen, Heng-Yu He, Yu-Xing Tong, Xue-Lin Chen

**Affiliations:** 1 College of Computer Science and Engineering, Jishou University, Jishou, 416000 China; 2 Faculty of Computing, Universiti Teknologi Malaysia, 80310 Skudai, Johor Malaysia; 3 UKM Graduate School of Business, Universiti Kebangsaan Malaysia, 43600 Bangi, Malaysia

**Keywords:** Sustainability, Information technology, Scientific data

## Abstract

To analyze the public opinion related to the employment situation, a combined approach is proposed to study the valuable ideas from social media. Firstly, the popularity of public opinion was analyzed according to the time series from a statistical point of view. Secondly, the feature extraction was carried out on the public opinion information, and the thematic analysis of the employment environment was carried out based on the Latent Dirichlet Allocation model. Thirdly, the Bert model was used to analyze the sentiment classification and trend of the employment-related public opinion data. Finally, the employment public opinion texts in different regions were studied based on the spatial sequence popularity analysis, keyword difference analysis. A case study in China is conducted to verify the effectiveness of proposed combined approach. Results shown that the popularity of employment public opinion reached the highest level in March 2022. Public opinions towards employment situation are negative. There is a specific relationship between the popularity of employment public opinion in different provinces.

## Introduction

Employment is the foundation of people’s livelihoods. Governments attaches great importance to employment issues and has always placed “employment stability” at the top of the “six stability” work^1^. Hence, it is imperative to identify the employment issues that the public emphasizes further help the decision-makers make better decisions. However, previous research on the employment situation is due to the statistical analysis of employment-related data and surveys^2^. There are defects such as different sources of data, low scientific validity, low efficiency, and biased opinion^3^.

With the above analysis, this study proposes a combined approach to explore the public’s attention and sentiment orientation to the employment situation by two text mining methods, including topic modeling and sentiment analysis. The distributed web crawler technology is utilizied in this study to capture a large amount of public opinion information about employment issues on social networks. Furtherly, combined methods of natural language processing (NLP) related technologies such as Term Frequency Inverse Document Frequency (TF-IDF) strategy and Latent Dirichlet Allocation (LDA) topic model as well as Bert model have been employed for text analysis, sentiment analysis, and so on^4^.

To sum up, this study attempts to answer the following questions:(1)What is the overall situation of blog posts about employment on Sina Weibo and Bilibili? (2) What is the sentiment trend of attention to employment situations over time? (3) What are the spatial differences of employment situations and their emotional differences?

The main innovation and contribution of the study include the following aspects:(1) The distributed web crawler technology has been utilized to collect public opinion on employment issues, which can manage URLs and set crawler parameters in the system to improve the accuracy of web page collection. (2) The hierarchical text mining method analyzes online public opinion for employment situations. Primarily, the sentiment analysis based on Bert with higher sentiment prediction accuracy is conducted, which can reduce the deviation caused by manual coding, improve the accuracy of sentiment prediction, make the analysis results more objective and practical, and expand the methodology of employment situation research.

The remainder of this paper is organized as follows. Section Related work includes related work and the principle of related methods. Section Research method and data illustrates the research methods and data, including distributed web crawler technology, keywords analysis (TF-IDF and LDA topic model), and Bidirectional Encoder Representations from Transformer (Bert) based sentiment analysis^5^. Section Results presents the results of the research. Section Discussion discusses the results in detail. Finally, Section Conclusion and limitations concludes the paper and outlines future directions.

## Related work

As a particular form of public social opinion, online public opinion can reflect the public's relatively influential and tendentious remarks and opinions on certain hotspots and focal issues in real life^6^. Public opinion analysis refers to using natural language, data mining, and other technologies to analyze and mine potential information in public opinion, which effectively supports various management and decision-making. It has been applied in all aspects of society as public opinion involves multiple fields and contains rich emotional information, such as stock market forecasting^7^, identification and management of urban traffic problems^8^, natural disaster loss estimation, policy reflection information collection^9^, etc. In addition, because the evolution of online public opinion is of great significance to social governance, many researchers have paid attention to the mastery and utilization of public opinion. The research on the use of public opinion was mainly reflected in mining data for trend analysis and prediction. For example, Bian et al.^10^, used public opinion analysis to explore the evolution of public sentiment under major public health emergencies; Li et al.^11^, conducted public opinion analysis on typhoons on Weibo to explore the public's emotional indicators of the development and changes of typhoons and predict disaster losses at the same time. Meng et al.^2^, used public opinion to analyze the emotional differences of people in different provinces on the three-child policy.

In recent years, due to various reasons, significant changes have taken place in the employment situation in China. Firstly, the pressure on economic development is increasing, and economic development in China faces rising supply and shrinking demand. Secondly, the structural contradiction faced by employment has not been fundamentally alleviated, as the coexisting situation of “difficulty in employment and recruiting”. Thirdly, the number of graduates has been increasing yearly, and it is expected to reach 10.76 million in 2023. Fourthly, some industries have yet to fully recover to the level before COVID-19, as the epidemic's impact on employment still exists, and the demand for work is still unstable. Finally, It is difficult for college graduates to study abroad, and offline campus recruitment is inconvenient. Therefore, to step up the implementation of various measures to stabilize enterprises, maintain the overall stability of the employment situation of college graduates, and promote economic development and social peace, it is necessary to conduct public opinion analysis on the employment situation to provide data support for accurately analyzing the employment situation for job seekers.

### Information collection and preprocessing

To realize large-scale data collection, most researchers use web crawler technology to collect the required information. The resource platforms for public opinion data are large news portals and social media platforms such as Sina Weibo, Bilibili video website, and so on, sharing information with multimedia forms such as pictures, text, and videos. Those platforms have many advantages, such as short information dissemination time, large and active users, extensive data sources, and colossal data volume. Large-scale active users in those platforms contribute to the quick response to hot events with broad coverage and a wide range of influence. For example, the Bilibili is mainly a gathering place for young people, especially college students. They can often express more rational and reasonable views on hot events, which aligns with general public cognition and positions. The collection of public opinion data was mainly realized through Python crawler technology (including the Python crawler network module “BeautifulSoup”, “requests”, “XML”, etc.) and the information collection tool “Octopus”. After the information was collected, a series of data preprocessing operations were carried out, including data cleaning, feature extraction, and construction of vector space. Firstly, the “is_null”, “drop”, “drop_duplicates”, and other methods in the “pandas” module were used to remove meaningless data such as missing, redundant, and cluttered data. Secondly, the Python-based Chinese word segmentation module, such as “jieba” was adopted to perform Chinese word segmentation and feature extraction. Finally, the processing results were formed into a matrix based on the vector space model.

### Popularity analysis

In quantifying the popularity of public opinion, previous researchers counted the number of comments on employment issues of each province on social media platforms. Then, the spatial sequence distribution map of the popularity of public opinion on employment issues was described. Further, the collected comment data was processed and analyzed to extract the keywords and conduct topic analysis.

#### Keyword analysis

Keyword analysis in public opinion analysis is fundamental to unraveling prevailing trends, sentiments, and critical issues within societal discourse. Various methodologies are employed for keyword analysis, each catering to specific research objectives. Natural language processing (NLP) techniques, including tokenization and part-of-speech tagging, enable the extraction of meaningful keywords. Additionally, machine learning algorithms, such as topic modeling and sentiment analysis, contribute to the identification of keywords that encapsulate prevalent themes and sentiments within textual data.

#### Thematic analysis

Thematic analysis in public opinion analysis is a qualitative research method aimed at identifying, analyzing, and reporting patterns or themes within textual data. In the context of public opinion, this approach helps researchers gain insights into the recurring topics, concerns, and sentiments prevalent in public discourse. It serves as a valuable tool for uncovering the nuances and intricacies of public sentiments on diverse issues.

### Sentiment analysis

Sentiment analysis plays a pivotal role in understanding public opinion. By integrating sentiment analysis with keyword analysis, researchers can identify keywords associated with positive or negative sentiments, providing nuanced insights into public emotions and perceptions.

To obtain the most suitable sentiment classification method for public opinion texts of employment and obtain the sentiment trend accurately, Bert, LSTM^12^, KNN^13^, and SVM^14^ methods were used in previous research.

## Research method and data

### Distributed web crawler technology

This research adopted the Nutch framework based on Hadoop, which employed the distributed web crawler Nutch to crawl public opinion information from the specified URL entry, performed data cleaning, deduplication, and noise removal based on the acquired public opinion information, and finally extracted plain text data as the data source for information preprocessing. Users can manage URLs and set crawler parameters in the system to improve the accuracy of web page collection.

This paper collected data on employment comment information released by netizens on Weibo and the BiliBili platform since 2019, which included the number of likes, IP location, and comment text information. Through analyzing the employment public opinion from collected data in terms of the three aspects of popularity, emotion, and theme.

### TF-IDF

Keyword analysis used TF-IDF (term frequency-inverse document frequency), a weighting technique widely used in data mining, as the extraction method of text feature words to evaluate the importance of words to a document. Among them, *TF* is the word frequency, which is the frequency of a specific expression in the document; *IDF* is the inverse text frequency index, which measures the overall importance of a comment. The calculation methods of *TF*, *IDF*, and *TF-IDF* are elucidated in the following Eqs. 1 to 3:

1 $${TF}_{\omega ,{D}_{i}}=\frac{count(\omega )}{|{D}_{i}|}$$

2 $${IDF}_{\omega }={\text{log}}\frac{N}{{\sum }_{i=1}^{N}I(w,{D}_{i})}$$

3 $${TF-IDF}_{\omega ,{D}_{i}}={TF}_{\omega ,{D}_{i}}*{IDF}_{\omega }$$

where *count (w) is* the number of occurrences of a word in a document, *|D*_*i*_*| is* the sum of words in the documents, *N is* the number of documents in the entire corpus.

### LDA

The operation process of the LDA probabilistic topic model^15^ is as follows:

- **Step 1**. For the topic *T*_*m*_, according to the Dirichlet distribution Dir (*β*), a word multinomial distribution vector *φ* on the topic was obtained ;
- **Step 2**. Obtaining the word number *N* of the text based on the Poisson distribution *P* ;
- **Step 3**. Obtaining a topic distribution probability vector *θ* of the text based on the Dirichlet distribution Dir (*α*), where *α* *was* the Dirichlet distribution parameter ;
- **Step 4**. For each of the *n* words of the text, *W*_*ni*_ randomly selected a topic *T*_*m*_ from the Multinomial (*θ*);
- **Step 5**. Selecting a word *W*_*n*_ from the multinomial conditional probability distribution Multinomial (*Φ*) of topic *T*_*m*_.

Through the above steps, the formula^15^ of the generation probability $(P({Wn}_{i}))$ of the *i*-th feature word in document *n* $({Wn}_{i})$ *was* obtained as Eq. 4:

4 $$P\left( {\omega_{{n_{i} }} } \right) = \mathop \sum \limits_{k = 1}^{T} P\left( {\omega_{{n_{i} }} {|}T_{{n_{i} }} = k} \right)P\left( {T_{{n_{i} }} = k} \right)$$

The generation probability formula of the LDA model^15^ was described as Eq. 5:

5 $$P\left( {\theta ,x,y{|}\alpha \beta } \right) = P\left( {\theta {|}\alpha } \right)\mathop \prod \limits_{n = 1}^{N} P\left( {x_{n} {|}\theta } \right)P\left( {y_{n} {|}x_{n} \beta } \right)$$

where *θ* represents the document layer, *x is* the feature words distribution of the hidden topic in the document, *y is* the feature word vector corresponding to the document, and the parameters *α* and *β are* used to generate vector *θ*, which is the probability distribution of the feature words of the hidden topic.

In addition, for the parameter *K* (number of topics) in the LDA model, Blei et al.^16^ used perplexity to measure the inaccuracy of the model's assignment to the topic model and determined the number of topics of the LDA topic, as shown in Eq. 6:

6 $$perplexity\left(T\right)=exp\left\{-\frac{\sum_{n=1}^{m}{\text{log}}p\left({W}_{n}\right)}{\sum_{n=1}^{m}{N}_{n}}\right\}$$

where *T* is the test set in the corpus, and there are *m* documents in total, ${N}_{n}$ represents the number of words in document *n*, ${W}_{n}$ represents the words in document *n*, and $p({W}_{n})$ represents the probability of word *W*_*n*_ generated in the document. Therefore, this paper used the perplexity coefficient to determine the optimal number of topics used in the experiment.

### Bert

**Figure 1:**
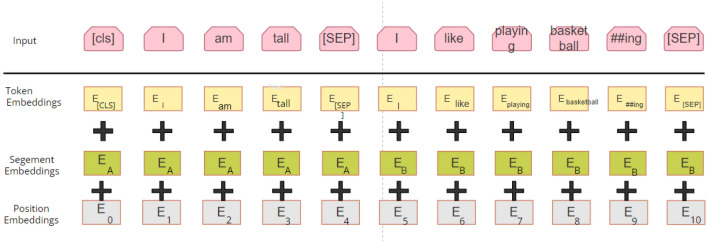
Diagram of three-layer Embedding processing mechanism.

Based on the analysis, it can be found that the Bert model can achieve better results compared with LSTM, etc. Hence, Bert is selected for sentiment analysis of employment public opinion texts. The pretraining process of the Bert model is as follows, and the process is shown in Fig. 1.

**Step 1**. Firstly, adding tokens (each word) to two consecutive sentences in the context.

Adding [cls] at the beginning of the sentence (for downstream classification tasks), adding [sep] between sentences and the end of the sentence (for sentence segmentation), and preparing two preceding and succeeding sentences with the same format but not in context;

**Step 2**. Randomly initializing a trainable segment embedding to make the model separate the upper and lower sentences. Meanwhile, assigning values of 0 and 1 to tokens in the preceding and succeeding sentences, respectively,  ensured that model could judge the starting and ending positions of the preceding and succeeding sentences.

**Step 3**. The vector corresponding to each word in the sentence was integrated into the text information based on the attention mechanism. It acquired the vector corresponding to the [cls] token in the final hidden layer. The hidden output of the model was finally obtained, and the dimension was calculated as Eq. 7:

7 $$X_{hidden} :\left[ {batch\_size,sep\_len,embedding\_dim} \right]$$

Taking out the vector corresponding to token [cls], where [cls] corresponded to the 0-th item of the *sep_len*:

8 $$cls\_vector = X_{hidden} \left[ {:,0,:} \right]$$

9 $$cls\_vector \in R^{batch\_size,embedding\_dim}$$

**Step 4**. Initializing a weight, completing the logistic regression from the embedding_dim dimension to 1, and then activating it with the sigmoid function to get the model's inference for the classification problem:

10 $$\hat{y} = sigmoid\left( {Linear\left( {cl{\text{s}}\_{\text{vector}}} \right)} \right){ }\hat{y} \subset \left( {0,1} \right)$$

## Results

### Popularity analysis based on time series

**Figure 2:**
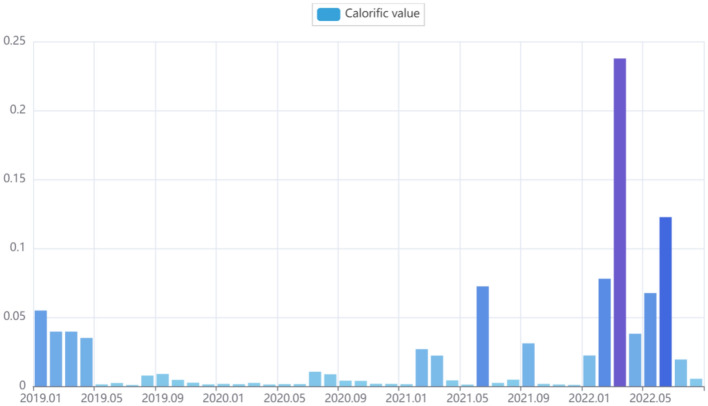
The popularity of employment public opinion based on time series.

An analysis of public opinion popularity in different periods was conducted to explore the impact on employment issues from different angles under the complex and ever-changing political, economic, educational, social, and humanistic background. Except for the heat analysis of the public opinion on the employment situation in the past three years, this paper used a visualization tool to construct a heat histogram based on time series. It analyzed the time characteristics of the public opinion information. As shown in Fig. 2, in the previous stage, public opinion enthusiasm from 2019 to 2020 showed a significant proportion only for the first four months of 2019, and the popularity could have been higher. In 2021, the popularity of public opinion began to fluctuate, and compared with the past period in May, it increased significantly. The total heat value in 2022 would be the highest in the past three years, and the heat value in March 2022 reached its peak, close to 1/4 of the total heat value in the past three years. It can be seen from the figure that the entire heat histogram is U-shaped, which shows that the discussion on the employment situation in 2022 is relatively hot. Most netizens have a solid response to the employment situation in 2022.

#### Keywords analysis

**Figure 3:**
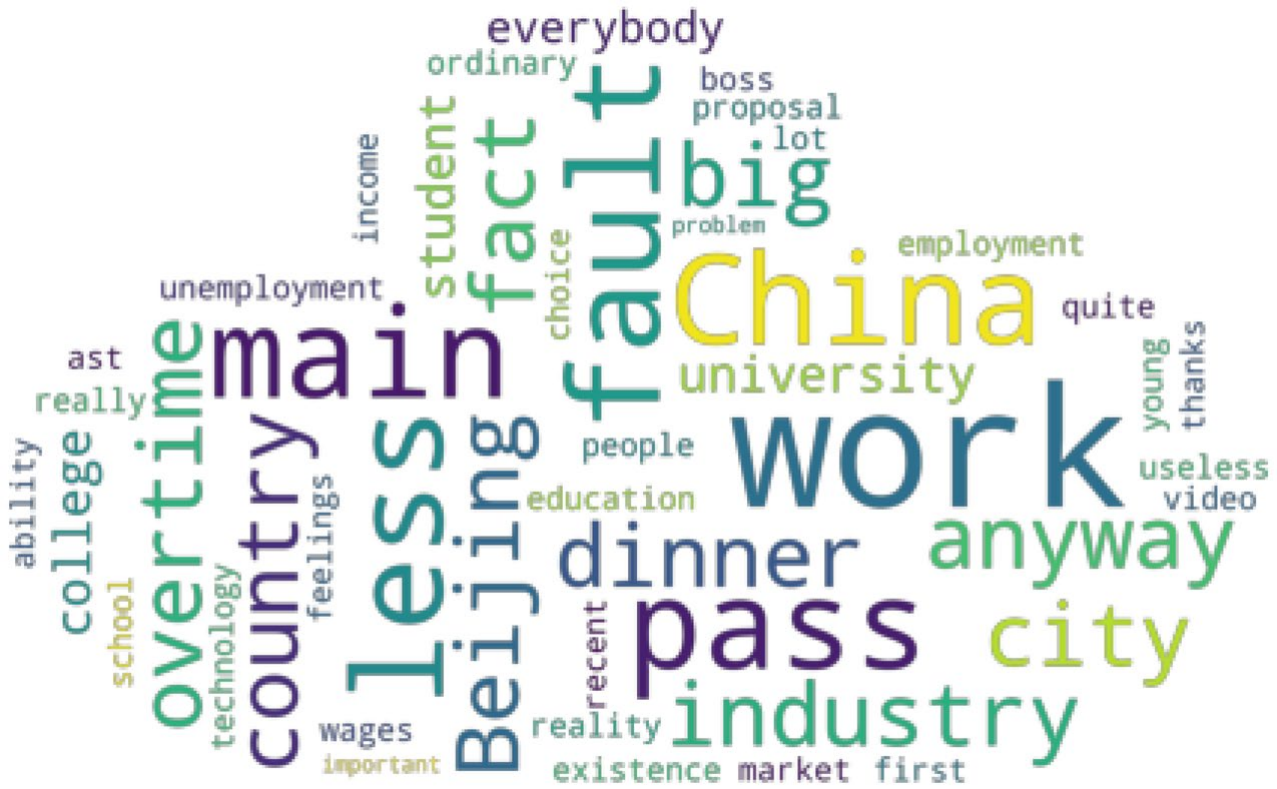
Word cloud of employment public opinion.

Based on the word frequency statistics of segmented text, the top 30 public opinion rankings in the comment data were obtained and analyzed. To clearly describe the comment emphasized by netizens, the word cloud of the comment data is shown in Fig. 3.

Words such as “985”, “educational degree”, “overtime”, “big city”, “college student”, “salary”, “university”, “meal”, and “unemployment” are eye-catching and frequently appear.

#### Thematic analysis

After the description of word frequency using the word cloud, the LDA thematic model was used for topic mining.

**Figure 4:**
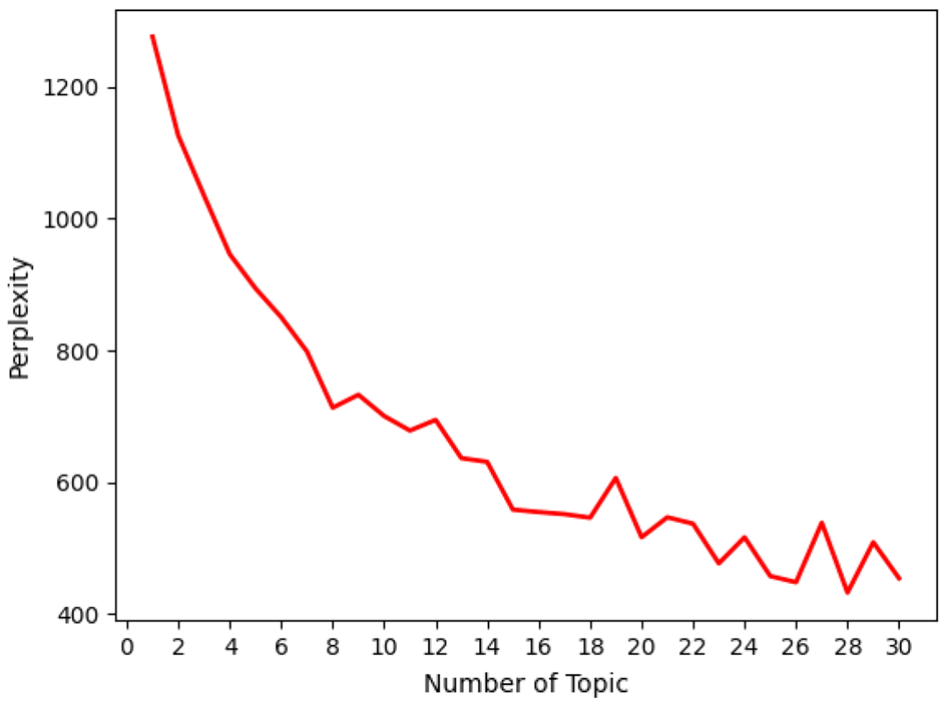
Perplexity graph of the LDA model.

First, an LDA thematic model was used to train the preprocessed data. It was necessary to choose the optimal number of topics to make the model show high performance on employment public opinion data. Hence, we used perplexity^17^ to select the number of topics for the LDA model and the smallest perplexity under a different number of topics as the optimal model. The number of topics selected in the experiment within the range of integers from 0 to 30. By calculating the perplexity under each number of topics, the perplexity curves under different numbers of topics were obtained, as shown in Fig. 4. It can be seen from the figure that the number of topics corresponding to the minimum value of perplexity is 28, indicating that the optimal number of topics is 28, which can make the LDA model show better performance on employment public opinion.

**Figure 5:**
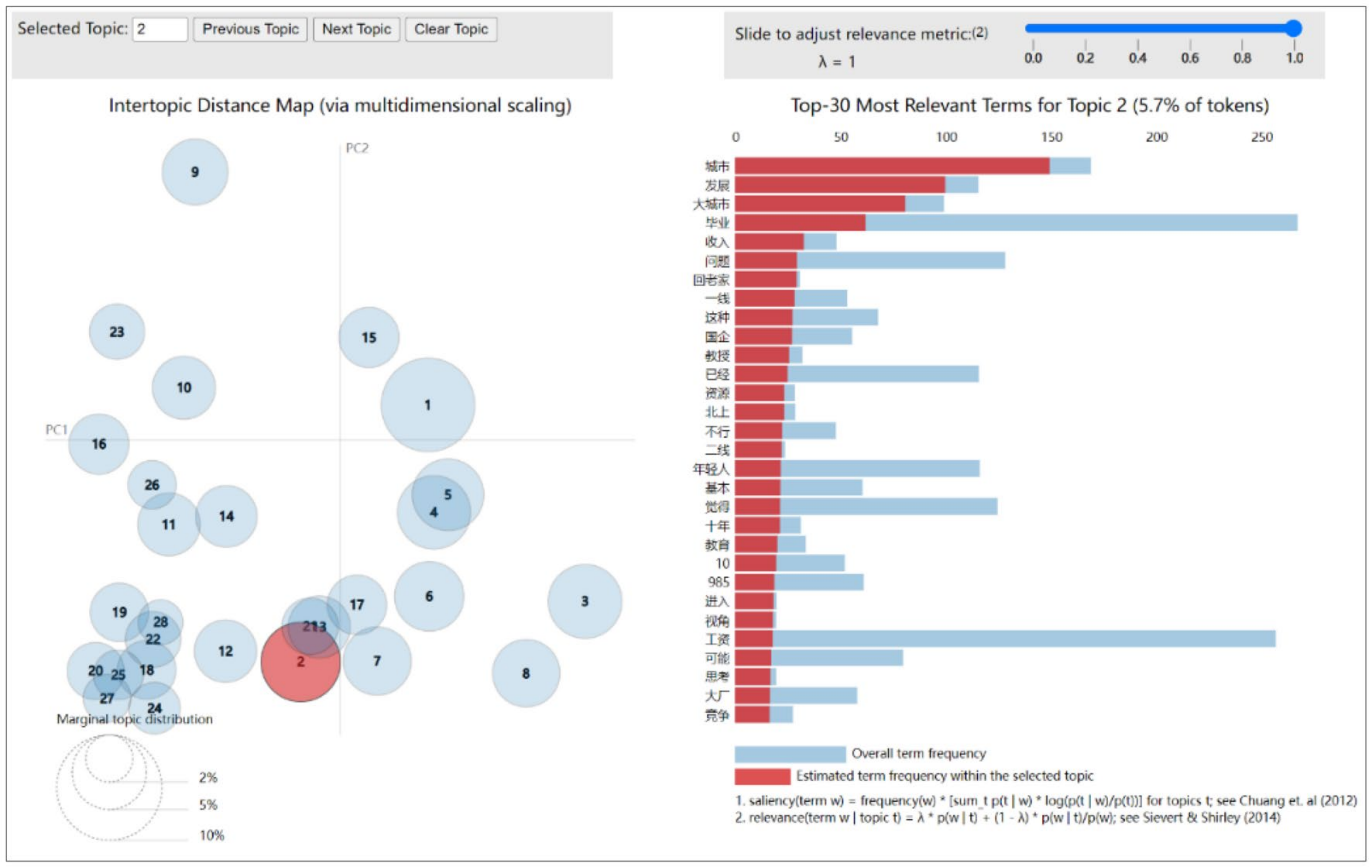
Theme mining and visual analysis with LDA model.

Next, this paper used the PyLDAvis visualization tool to visually analyze the potential topic information mined by the topic of the LDA model and obtained the effect diagram shown in Fig. 5. The top keywords in topic 1 included “education,”, “ability”, “learning”, and “university”. Combined with the sentiment analysis results above, netizens had many difficulties regarding education requirements in today's employment issues. The focus of theme 2 shows the public's preference for cities, words such as “big city” and “northbound” are more frequent, followed by “second-tier”. The feature words of topic 3 are mainly employment environment, including “graduation”, “internship”, “company”, “society”, “Foxconn”, “monthly salary”, “job”, “programmer”, “offer”, and other related words.

### Sentiment analysis

**Figure 6:**
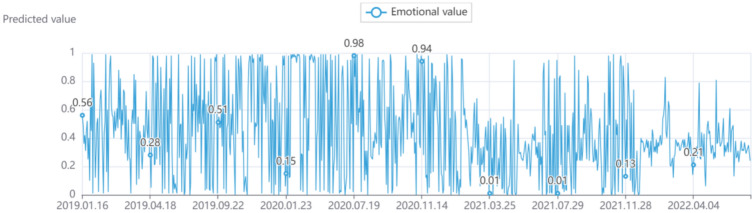
Sentiment trend analysis of employment public opinion.

The sentiment analysis results of each comment were obtained based on the Bert algorithm^18^. Partial data from the three transition zones are illustrated in Fig. 6. In this paper, 70% of the data was set as the training set and the rest as the testing set. It is evident from the figure that more individuals possess negative attitudes than positive attitudes (sentiment analysis results suggest that 0.50 means that the emotion has no emotion, which is a neutral state, 0.50 to 1.00 is a positive state; the more significant the value, the more positive the emotion; 0.00 to 0.50 is a negative state, the smaller the value, the more negative the emotion).

The numerical fluctuations fluctuated wildly, gradually slowed down over time, and finally reached a low point in 2022 and remained stable. From a local point of view, the image results were roughly divided into three transition zones: December 2020, October 2021, and early 2022. Before December 2020, the emotional changes of netizens generally tended to be positive. Between transition zones, emotional changes fluctuated rapidly between positive and negative. After the beginning of 2022, the predicted values showed that sentiment changes were roughly between negative and neutral.

### Spatial difference analysis

Public opinion research found that people in different regions paid different attention to employment issues^18^. To study the spatial differences in employment issues and analyze the public opinion information of different provinces more accurately, separate studies of network public opinion in different provinces were carried out.

#### Popularity analysis of spatial difference

**Figure 7:**
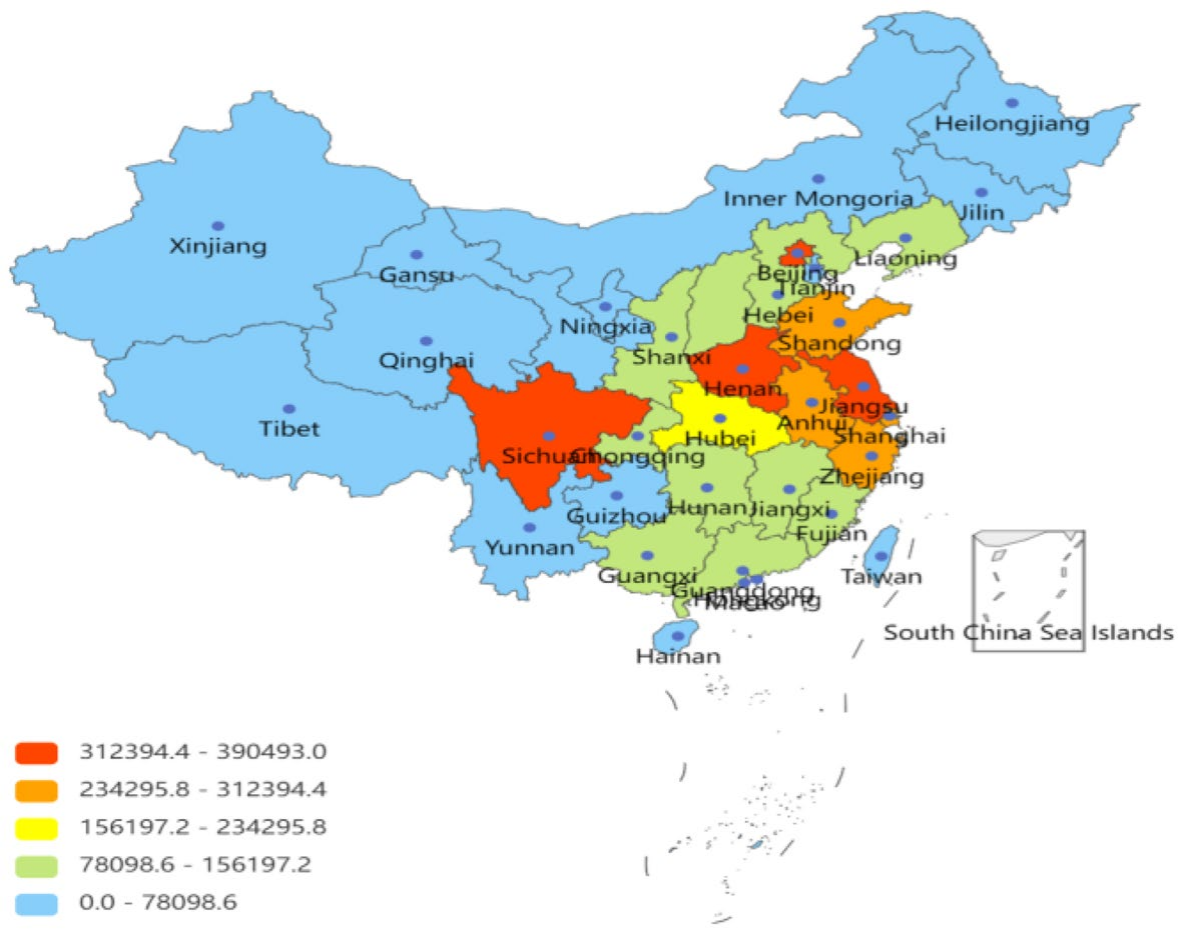
The popularity of public opinion based on spatial sequence.

To study the differences in employment public opinion in different provinces and regions, this paper classified the crawled IP data^19^. It used the popularity analysis method mentioned above to obtain each province's public opinion heat value. To better describe the differences in the popularity of public opinion about employment issues in different regions, this paper used the “Piecharts” module to construct the public opinion heat graph, as shown in Fig. 7.

**Figure 8:**
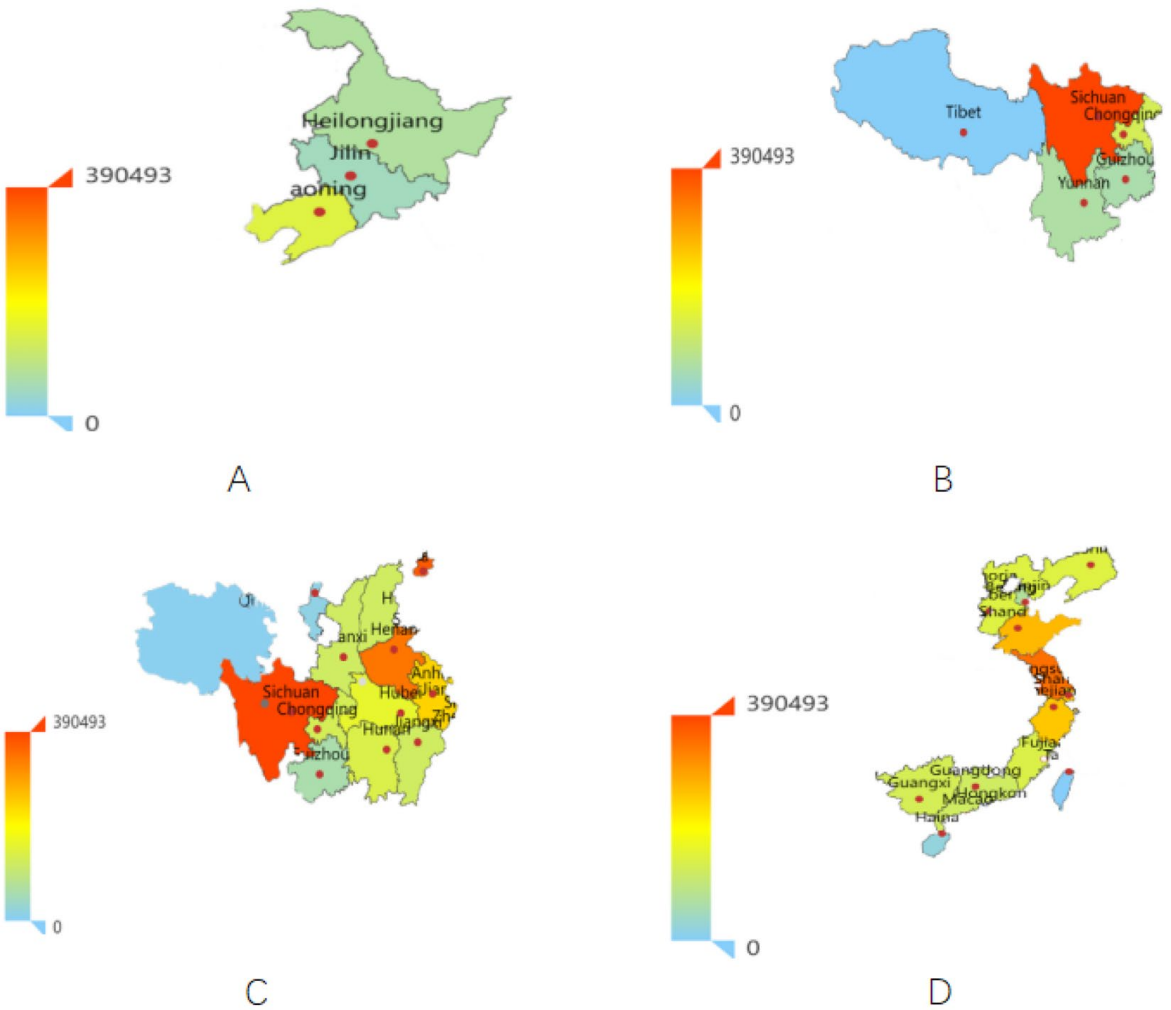
The popularity of public opinion based on different regions.

Figure 7 shows that the most popular coastal provinces, such as Jiangsu, Shanghai, and Shandong, are the most concerned with employment public opinion, followed by provinces with large populations, such as Sichuan, Beijing, and Henan. To analyze the spatial distribution of heat values, as shown in Fig. 8, the country is divided into four regions, northeast-southwest and coastal-inland regions, for pairwise comparisons.

#### Keyword analysis of spatial difference

To facilitate further exploration of differences in public opinion in different provinces, this paper took the comment data of the top eight provinces for text analysis and keyword extraction^20^. Then, the keyword list of public opinion on employment issues in different provinces was obtained, as shown in Table 1.

**Table 1:** Keywords of public opinion in different provinces.

Province	Guangdong	Sichuan	Henan	Jiangsu	Zhejiang	Shanghai	Beijing	Hunan
TOP1	HR	normal	Undergraduate	Educate	Nation	Postgraduate	HR	Research
TOP2	Industry	Education	Work	Master	Graduate	Dividend	Elderly	Postgraduate
TOP3	Effort	Enterprise	Young	Postgraduate	High school	Parents	Education	Student
TOP4	Nation	Scholarship	Company	Dormitory	Graduate	Private enterprise	Company	Resource
TOP5	Technology	family	Bachelor of Science	Resource	Enterprise	Enrollment expansion	Elderly	Technology

Employment is the foundation of people's livelihood and the essential prerequisite and way to improve people's living standards and quality of life. As a fundamental issue related to the national economy and people's livelihood, employment is closely related to the “economy”, “politics”, “education”, “society”, etc. Compared with the overall keywords, it can be intuitively seen from Table 1 that the public opinion directions of the keywords in different provinces are more precise, which can better reflect the concerns of people in different regions. For example, netizens in Guangdong paid more attention to the general environment of employment, which was reflected in words such as “industry”, “country”, and “technology” in the vocabulary. Meanwhile, netizens in Beijing paid more attention to the differences in employment among people of different age groups, mainly focusing on “old age” and related information. There were different focus points on employment issues of different provinces. Correspondingly, the government's policy work and employment subsidies should also target the focus of different provinces.

From the overall keyword distribution, it can be found that netizens' concerns about employment include “education”, “national policies”, and “enterprises”. Moreover, it can be seen from the table that most provinces contain keywords related to education.

In summary, in the study of online public opinion on employment issues, the public opinion data of each province were studied separately, and the focus of each province differed from that of other provinces. Research can grasp the development direction of public opinion more deeply, tap important potential information related to public opinion, and provide data reference for the government to issue relevant decisions.

## Discussion

### Discussion of results on RQ1

The first four months of 2019 have had a comparative proportion because the four months were during the spring recruitment stage in China. Since the explosion of COVID-19 in January 2020, all walks of life have been in a downturn under the epidemic's impact. The entire heat histogram is U-shaped and can be explained by combining the social background; the fight against COVID-19 in 2021 has achieved outstanding victory. The spring recruitment of college graduates began in February 2022 and reached its peak in April 2022. In addition, combined with the details of opinion content, it was found that college students were worried about the uncertainty of the future and also pessimistic about the employment environment.

The following conclusions are given from the keywords analysis and combined with the text content.

1. Under the current employment background, college students are most concerned about employment issues, and many are worried about the educational threshold for employment.
2. In today's society, where the economy and society are developing, and the pressure of life is increasing daily, the cost of living in big cities is increasing, and the phenomenon of overtime work by employees has gradually become common.
3. Today's complicated employment situation has made some employed people worry that they may lose their jobs and have uncertainty about their future work and life.

### Discussion of results on RQ2

Understanding public sentiment orientation toward employment is critical to addressing employment issues. Figure 6 reflects the emotional attitudes of netizens towards employment topics in different periods. Excluding some results with little error, the psychological change process of netizens can be linked to the actual employment environment. The sentiment orientation obtained from this study has shown that 73% of the Chinese people studied have a negative sentiment about employment issues. The reason may be the following aspects.

First, in 2022, college graduates exceeded 10 million for the first time, and many companies were laying off employees. Second, under the dual influence of COVID-19 and the complex international situation, more and more international students have chosen to return to China for employment, further increasing the competitiveness of domestic employment. To an extent, the problematic employment situation will also affect the mentality of netizens when facing employment and increase the appearance of negative emotions in public opinion. The government's implementation of employment policies and scientific guidance to the employed population can significantly stimulate the enthusiasm of public opinion.

### Discussion of results on RQ3

#### Popularity analysis between different regions

Comparing and analyzing the heat value results in Figs. 7 and 8 shows that the netizens in the southwest region are more involved in public opinion on employment issues than those in the northeast region. In addition, the employment public opinion heat value is higher in the eastern coastal cities and the inland provinces with large populations. The following reasons can explain it.

1. Economic development differences. Coastal provinces generally have more developed economic systems, attracting more foreign and domestic capital investment. This has led to a more diversified and modernized industrial structure in coastal areas, providing more employment opportunities. On the contrary, some inland provinces may face constraints due to geographical and transportation conditions, necessitating efforts for economic catch-up, and consequently, offering relatively fewer employment opportunities.
2. Export-oriented economy. Coastal provinces usually rely more on foreign trade and the introduction of foreign investment, so they pay more attention to attracting enterprises and foreign investment and improving employment levels. By developing an export-oriented economy, these provinces can better integrate into the global industrial chain and create more job opportunities.
3. Speed of urbanization. Urbanization in coastal provinces is relatively fast. Urbanization has driven the development of the service and manufacturing industries, providing more employment opportunities for urban residents. In contrast, the urbanization process in some inland provinces may need to catch up, and employment opportunities may be limited.
4. Educational resources. Coastal provinces, including universities and vocational training institutions, generally have better educational resources. This makes the labor force in coastal areas more competitive and adaptable to market demand, making it easier to find employment opportunities.
5. Policy orientation. The Chinese government may focus more on developing coastal areas in its economic policies and promote local economic growth and job creation through policy measures.

It should be noted that although coastal provinces are relatively more concerned about employment issues, at the national level, the Chinese government has been committed to achieving comprehensive, balanced, and sustainable development, promoting economic growth in inland areas, narrowing regional development gaps, and improving the standard of living for people across the country.

#### Keyword analysis of spatial difference

By analyzing the keywords of employment issues in the eight provinces, we can find some differences in the employment issues in each province. The following is a brief analysis of the concerns of each province.

As a relatively developed province in China, Guangdong has diversified industrial development, so human resources, industry, and technology are more prominent concerns. Guangdong's economic structure has a significant demand for talent at different levels, so it pays attention to talent training and technological innovation.

Sichuan may pay more attention to ordinary workers, education and training, enterprise development, etc. Due to Sichuan's geographical and economic characteristics, it may be more concerned about primary education and the employment of ordinary workers.

Henan's focus is mainly on talent training and employment at the undergraduate level, which may reflect the demand for highly educated talents. Concerns of young people and companies may be related to economic development and industrial structure.

Jiangsu is an economically developed province focusing mainly on higher education, master's degree training, and dormitory resources. This may be related to Jiangsu Province's technological innovation and high-skilled talent cultivation.

Zhejiang pays attention to national-level employment policies and also pays attention to the employment issues of graduates. Enterprise and technology concerns may be related to the private economy and technology industry in Zhejiang Province.

Shanghai focuses on research, dividends, and other issues related to high-tech industries, households, and private enterprises. Enrollment expansion may be related to Shanghai's university development and talent introduction policies.

Beijing pays attention to human resources, employment issues for the elderly, companies, and resources. This may reflect the agglomeration of high-tech industries and large enterprises in Beijing, as well as concerns about the employment of the elderly and the employment needs of enterprises.

Hunan's focus on research, graduate students, and technology may be related to Hunan Province's university development and scientific and technological innovation policies. The concerns about students and resources may be related to Hunan's distribution of education and resources.

The distribution of these keywords reflects the different concerns about employment issues in each province. These concerns are mainly affected by factors such as each province's economy, education, and industrial structure. Different provinces' economic characteristics and social needs have led to differences in the focus on employment issues.

## Conclusion and limitations

The analysis of online public opinion on the employment situation is of great significance for guiding government decision-making and public opinion control^20^. This study combined the TF-IDF, LDA, and Bert models to explore the hot thematic and sentiment orientation. Then, core questions have been answered, such as the overall situation of blog posts about employment issues, the trend of the attention to employment situations over time, and the spatial difference analysis of employment situation sentiment orientation. The inclusions are as follows.

1. The popularity analysis based on time series revealed that the COVID-19 outbreak affect netizens' emotions toward employment. With China's victory in fighting the epidemic, some affected industries has gradually recover, providing more employment opportunities. In addition, the Chinese government has adopted a series of policies to support stable employment for enterprises, including fiscal stimulus, tax and fee reductions, and employment assistance. Hence, the heat value increased in May of 2021. However, the The epidemic is intermittent in China, which caused the fluctuation of heat value.
2. Many netizens have a high degree of participation in employment-related topics. For employment issues, most netizens focus on academic requirements and the status of social needs. Netizens attach great importance to employment issues and look forward to relevant follow-up measures.
3. There are apparent differences in netizens' attention to employment issues in different provinces. Employment issues are of higher concern in economically developed areas such as coastal areas but lower in remote and economically underdeveloped inland areas. More graduates focus on better-paying jobs due to the continuously increasing talent saturation and high living costs in these regions, exacerbating the contradiction between supply and demand. Different provinces have differences in the focus of employment issues due to their different political, economic, and educational backgrounds. When studying each province's public opinion, it is necessary to identify the keywords unique to each province and analyze each province's employment policies and social needs.

The limitations of this study and future research directions are mainly described as follows.

1. The data source in this article is mainly public opinion information about employment generated in Weibo and Bilibili, so the data source platforms need to be revised. In future research, as much as possible, more current mainstream network information platforms should be incorporated into the data collection sources.
2. The object of data collection in this paper is mainly for plain text data, and other types of data information, such as pictures and videos, are also of great value for public opinion analysis. Therefore, follow-up researchers can collect information on various data types and conduct comprehensive analysis and processing.
3. The period of data information selection is relatively large. However, with time increasing, the value of data information in specific periods for analysis and research will decrease. Therefore, neglecting to weigh information from different periods will also affect the final research results. In the future, we will assign weights to the data information in different periods to improve the accuracy of the research results.

## Data Availability

The datasets generated during and analyzed during the current study are available from the corresponding author upon reasonable request.

## References

[1] Li, H. N. Precisely implement policies to tap the potential employment market. China Economic Times, 2022-07-25(001).

[2] Meng FS, Zhong H, Shi SC, Xie ZK (2022). Analyzing public opinion on three-child-policy with sentiment classification and keyword extraction. Data Anal. Knowl. Discov..

[3] Guo Y, Gong YY, Zhang Q, Huang XJ (2018). Retweet behavior prediction using topic model. Chin. J. Inf..

[4] Wang SP, Peng Y, Wang J (2014). Application of text clustering based on LDA in network public opinion analysis. J. Shandong Univ. (Nat. Ed.).

[5] Wu M, Long R, Chen F (2023). Spatio-temporal difference analysis in climate change topics and sentiment orientation: Based on LDA and BiLSTM model. Resour. Conserv. Recycl..

[6] Wang N, Li HR, Tan SR (2022). Predicting public opinion reversal based on evolution analysis of events and improved KE-SMOTE algorithm. Data Anal. Knowl. Discov..

[7] Ma YY, Liu YZ, Liu CL, Zhang TJ (2022). Chinese investors’ multi-perspective sentiment analysis and its role in stock market forecasting. J. Northeastern Univ. (Nat. Sci. Ed.).

[8] Liu C, Guo L, Fan ZY (2022). Study on identification and governance countermeasures of the traffic problems in metropolis based on online public opinion: A case study of Wuhan city. Urban Issues.

[9] Song HS, Zhang WP, Yang C, Zhang SB (2022). Characteristics of online public opinion of the “doube reduction” policy, public concern and government responses: The big data analysis based on the national E-government platform. Primary Educ..

[10] Bian XH, Xu T (2022). Evolution of public sentiment during COVID-19 pandemic. Data Anal. Knowl..

[11] Li SP, Zhao F, Zhou YQ, Tian XL, Huang H (2022). Analysis of public opinion and disaster loss estimates from typhoon based on Microblog data. J. Tsinghua Univ. (Nat. Sci. Ed.).

[12] Zhang XL, Zhang DF, Liu W, Yang Q, Guo YY, Ren YH, Fan ZX (2022). Hourly concentration prediction of PM_2.5_ based on multi-channels long short term memory. Environ. Sci. Res..

[13] Wu M, Long R, Chen H (2023). The spatial difference of multi-layer climate change information flow and network construction: A comparison of “dual carbon” scenarios. J. Clean. Prod..

[14] Wen Y, Zhao X, Li X, Zang Y (2023). Explaining the paradox of world university rankings in China: Higher education sustainability analysis with sentiment analysis and LDA topic modeling. Sustainability.

[15] Zhang MH, Wang HL, Zhou GD (2011). An automatic summarization approach based on LDA topic feature. Comput. Appl. Softw..

[16] Blei D, Ng A, Jordan M (2001). Latent dirichlet allocation. J. Mach. Learn. Res..

[17] Yang Y, Jiang KZ, Yuan MJ, Hui LX (2022). Selecting optimal LDA numbers to identify news topic. Data Anal. Knowl. Discov..

[18] Wu Z, Zhang Y, Chen Q (2021). Attitude of Chinese public towards municipal solid waste sorting policy: A text mining study. Sci. Total Environ..

[19] Chakraborty K, Bhattacharyya S, Bag R (2020). A survey of sentiment analysis from social media data. IEEE Trans. Comput. Soc. Syst..

[20] Ye S, Zhou K, Zain AM (2023). A modified harmony search algorithm and its applications in weighted fuzzy production rule extraction. Front. Inf. Technol. Electron. Eng..

